# A Machine Learning Approach to Voice-Based Parkinson Disease Screening Using Multiview Spectrogram and Speech Recognition Features: Diagnostic Study

**DOI:** 10.2196/94063

**Published:** 2026-06-11

**Authors:** Arifa Zahir, Jaehong Yu, Jin-Sun Jun, Kiwon Park, Ryul Kim, Hyundoo Jeong

**Affiliations:** 1 Department of Biomedical and Robotics Engineering, Incheon National University, 119 Academy-ro, Yeonsu-gu, Incheon, 22012, Republic of Korea, 82 32-835-8677; 2 Department of Industrial and Management Engineering, Incheon National University, Incheon, Republic of Korea; 3 Department of Neurology, Kangnam Sacred Heart Hospital, Hallym University College of Medicine, Seoul, Republic of Korea; 4 Department of Neurology, Seoul Metropolitan Government-Seoul National University Boramae Medical Center, Seoul National University College of Medicine, Seoul, Republic of Korea

**Keywords:** Parkinson disease, voice-based screening, multiview spectrogram, deep learning, multiview learning, automatic speech recognition

## Abstract

**Background:**

Parkinson disease frequently manifests early vocal impairment, motivating the development of noninvasive and scalable digital screening tools.

**Objective:**

This study proposes a multiview spectrogram-based deep learning framework integrating recognition-aware context for Parkinson disease detection from voice recordings.

**Methods:**

Voice recordings from 203 participants (121 with Parkinson disease and 82 healthy controls) were collected prospectively. Three spectrogram representations (Mel, short-time Fourier transform, and constant-Q transform) were extracted and processed through parallel convolutional neural network branches. A recognition ratio (RR) feature vector derived from automatic speech recognition transcript agreement was optionally fused with spectrogram embeddings. Models were evaluated using strict subject-wise 5-fold cross-validation.

**Results:**

Multiview spectrogram recognition-aware Parkinson detection network achieved a mean test accuracy of 86.9% (SD 25.2%) using 3-view spectrogram fusion, improving to 97.4% (SD 5.7%) when incorporating the RR feature. RR integration reduced the false negative rate by approximately 84.5%, substantially improving sensitivity in screening-oriented settings.

**Conclusions:**

Combining multiview spectrogram learning with recognition-aware context significantly enhances voice-based Parkinson disease classification under leakage-free evaluation. These findings support the potential of this approach for noninvasive screening in structured recording settings, while further validation in diverse real-world environments is needed.

## Introduction

Parkinson disease is a neurological condition that progresses over time and manifests with various motor and nonmotor symptoms [1]. There is an increasing need for accurate health informatics systems to support its identification because early detection can improve clinical outcomes and enable timely intervention. Automated screening tools can also reduce the workload for clinicians and support large-scale monitoring [2,3]. Among accessible sensing modalities, vocal impairment is one of the most prevalent early symptoms, and voice-based assessment has become an important direction for Parkinson disease identification research [4-6].

Speech impairments are highly prevalent in Parkinson disease and may involve both speech production and language-related difficulties [7]. Clinically, these impairments encompass reduced vocal loudness (hypophonia), imprecise articulation, a monotone or breathy voice quality, and festinating speech, all of which reflect the combined effects of motor rigidity, bradykinesia, and reduced respiratory drive on the phonatory system [8]. Increasing evidence suggests that speech and language abnormalities can emerge prior to prominent motor signs and formal diagnosis [9]. Consequently, speech and language pathology has been recognized in clinical guidelines as a crucial component of Parkinson disease care from the early stage of diagnosis [8]. Recent research highlights the potential of objective acoustic markers to identify Parkinson disease in early or prodromal stages, creating a therapeutic window for early intervention [10,11]. Speech and language characteristics can also serve as surrogate markers for tracking disease progression [12], and distinct patterns of impairment have been linked to Parkinson disease subtypes and related movement disorders, supporting differential diagnosis using voice biomarkers [13-15].

Earlier studies in Parkinson disease voice analysis primarily relied on handcrafted acoustic features such as jitter, shimmer, harmonics-to-noise ratio, and Mel-frequency cepstral coefficients, combined with supervised classifiers such as support vector machines, random forests, or gradient-boosted trees [16,17]. More recent studies extend this paradigm through improved feature selection, interpretable machine learning, and cross-corpus evaluation [17-19]. Despite these advances, many approaches still depend on manually engineered descriptors and may underuse the richer structure present in the speech signal. To better capture Parkinson disease–related phonatory and articulatory cues, many groups transform audio into time-frequency representations and apply convolutional neural networks (CNNs) or transformer models directly to spectrograms [20,21]. Spectrogram features combined with artificial intelligence models have achieved strong performance for early diagnosis, supporting the clinical viability of spectrogram-driven approaches [22].

Despite these advances, most models still operate on a single spectrogram view. This is a limitation because different time-frequency representations emphasize complementary aspects of the signal: the short-time Fourier transform (STFT) provides a linear frequency axis with uniform resolution, Mel spectrograms approximate human auditory spacing and emphasize low-to-mid frequencies, and the constant-Q transform (CQT) yields a logarithmic frequency grid that can better represent harmonic and pitch-related patterns relevant to the vocal tremor and dysphonia observed in Parkinson disease. Prior research works in audio and biomedical sound classification show that fusing multiple spectrogram representations can yield more discriminative embeddings than any single representation alone [23-25]. For Parkinson disease speech, however, multispectrogram fusion remains underexplored [26,27].

A second challenge is data scarcity and overfitting. Even recent cohorts often include only a few dozen to a few hundred participants, and many studies rely on highly reused benchmark datasets. Previous studies have emphasized concerns, such as participant overlap between training and evaluation sets and optimistic performance estimates, that hinder clinical translation [16,28]. To address these issues, our study emphasizes strict participant-wise separation, controlled preprocessing, consistent model comparisons, and ablation-based analysis.

Finally, global context–based and recognition-based voice features are rarely integrated into voice-based Parkinson disease classification models. Digital biomarker research increasingly highlights the value of multimodal and metadata-informed fusion for stabilizing predictions [29-31]. For voice, a compact recognition-based feature vector can be derived from the same speech recording, referred to here as the recognition ratio (RR). Intuitively, the RR measures how accurately an automatic speech recognition system can transcribe what a participant said: a high RR indicates clear, intelligible speech, whereas a low value reflects speech that is difficult for the system to parse—consistent with the articulatory and phonation difficulties clinically observed in Parkinson disease [7]. People with Parkinson disease may experience difficulty in clearly pronouncing sentences, which can reduce speech intelligibility and articulation clarity. As a result, the RR can serve as a global, recording-level indicator of intelligibility, providing complementary context to the local spectro-temporal patterns learned from spectrograms. Such recognition-aware integration strategies remain uncommon in voice-based Parkinson disease classification models. From a clinical perspective, these speech abnormalities are directly reflected in acoustic representations of voice. Time-frequency spectrograms can capture changes in vocal intensity, pitch stability, and articulation patterns associated with Parkinson disease speech impairment, while the RR provides a complementary measure of speech intelligibility at the utterance level [7,8,22]. Therefore, combining multiview spectrogram features with recognition-aware information allows the model to capture both local acoustic patterns and global intelligibility deficits that are clinically relevant in Parkinson disease.

In this study, we propose a multiview spectrogram-based deep architecture for noninvasive Parkinson disease screening from voice. From each recording, we derive 3 normalized spectrogram types (Mel, STFT, and CQT) and feed them into parallel CNN branches whose outputs are concatenated. We further introduce a low-dimensional RR vector computed from the same audio but outside the image domain and concatenate it with the spectrogram-based representation to provide global context. Model evaluation is performed under participant-wise cross-validation with strict separation of speakers between folds.

The contributions of this study are 3-fold:

1. A multibranch CNN architecture is introduced that exploits Mel, STFT, and CQT spectrograms through parallel branches, and multiview feature concatenation is shown to improve performance compared with single-view models and recent spectrogram-based baselines.
2. The effect of recognition-based features is evaluated by integrating a low-dimensional RR vector as lightweight global context. Performance gains are quantified using accuracy, precision, recall, *F*_1_-score, and area under the receiver operating characteristic curve, demonstrating that contextual information can stabilize Parkinson disease voice classification.
3. A unified benchmark comparison is presented between classical acoustic feature machine learning baselines and spectrogram-based CNNs using a consistent participant-wise protocol. Comparison with recent state-of-the-art deep learning methods is also provided to contextualize the approach within the broader literature.

## Methods

### Dataset

A total of 203 participants were enrolled, including 121 individuals diagnosed with Parkinson disease and 82 healthy controls. The Parkinson disease group comprised 53 female participants and 68 male participants, while the healthy control group comprised 50 female participants and 32 male participants. The mean age was 68.7 (SD 8.9) years in the Parkinson disease group and 65.3 (SD 9.8) years in the healthy control group. Disease severity was assessed using the MDS-UPDRS-III (Movement Disorder Society–Sponsored Revision of the Unified Parkinson Disease Rating Scale) score (range 3‐55; mean 26.7, SD 10.6, and median 25.5, IQR 19.0-34.0). Of these, 192 participants (112 with Parkinson disease and 80 healthy controls) had complete and usable audio recordings across all required speech tasks and were included in model training and evaluation. Eleven participants (9 with Parkinson disease and 2 healthy controls) were excluded due to missing recordings or data quality issues identified in the source dataset. Table 1 summarizes participant characteristics.

Recordings were collected in a hospital inspection room using a Samsung Galaxy Tab S7 FE positioned approximately 30 cm from the participant’s mouth. Audio was recorded in MP3 format at 48 kHz (32-bit) and converted to WAV format prior to preprocessing. The speech protocol included 2 tasks: sustained vowel phonation of /a/ (Task 1) and reading 20 sentences comprising 10 nonmeaningful and 10 meaningful sentences (Task 2). The 20 sentence-reading items are indexed as test cases 1‐20; test cases 1‐10 correspond to nonmeaningful utterances, and test cases 11‐20 correspond to meaningful sentences.

**Table 1.** Summary of study population characteristics.

Characteristic	PD^a^ group (n=121)	HC^b^ group (n=82)
Sex		
Female, n	53	50
Male, n	68	32
Age (y), mean (SD)	68.7 (8.9)	65.3 (9.8)
MDS-UPDRS-III^c^ score		
Range	3–55	—^d^
Mean (SD)	26.7 (10.6)	—
Median (IQR)	25.5 (19.0-34.0)	—

a PD: Parkinson disease.

b HC: healthy control.

c MDS-UPDRS-III: Movement Disorder Society–Sponsored Revision of the Unified Parkinson Disease Rating Scale.

d Not applicable.

### Audio Preprocessing

Preprocessing was performed for 2 components: (1) time-frequency spectrogram representations used as CNN inputs, and (2) RR features used as the recognition-aware component. Each recording was standardized in the time domain (resampling to a fixed sampling rate, mono conversion, and conservative trimming), converted into 3 time-frequency views (Mel, CQT, and STFT), log-compressed, normalized using training-fold statistics, and resized to 128×128 grayscale images for network input. Detailed spectrogram normalization equations are provided in Multimedia Appendix 1. All normalization parameters were estimated using only the training subset within each fold and applied unchanged to the validation and test subsets to prevent data leakage.

Audio files were loaded from WAV format and processed using librosa [32]. The Mel representation used 128 Mel bands, a fast Fourier transform size of 2048, Hann window length of 2048, and a hop length of 512. The CQT used a hop length of 512, a minimum frequency of approximately 32.7 Hz, 12 bins per octave, and 84 frequency bins. The STFT used a fast Fourier transform size of 2048, Hann window length of 2048, and a hop length of 512. Modality-specific normalization (min-max for Mel, *z*-score for CQT, and robust scaling for STFT) was applied fold-wise using training statistics only (Multimedia Appendix 1).

To obtain the recognition-aware feature, we used RR, which provides an intuitive measure of how well an automatic speech recognition system understands the participant’s speech. Higher RR values indicate clearer and more intelligible speech, whereas lower values reflect reduced speech clarity.

Speech intelligibility is frequently compromised in individuals with Parkinson disease due to the progressive deterioration of motor control underlying speech production [33,34]. This deterioration often results in decreased articulatory precision, particularly evident when producing phonetically complex utterances that demand fine-grained neuromuscular coordination [35,36]. To capture this clinically relevant dimension of speech, we incorporate RR as a complementary feature. The sentence-reading task used in this study was designed to encompass both contextually meaningful sentences and phonetically demanding word sequences, providing a suitable basis for RR to reflect the degree of articulatory impairment.

The RR was computed from the sentence-reading task by comparing the target transcript with the automatic speech recognition output using a normalized edit distance:


(1) $$RR(\%)=100\left(1-\frac{d_{edit}(y_{target},\;y_{rec})}{\left|y_{target}\right|}\right),\;RR(\%)\in [0,100]$$

where *d*_edit_(·*,*·) is the Levenshtein distance and |*y*_target_| is the number of characters in the target string. The RR is computed independently for each recording using only its corresponding speech input and target transcript, without using any information from other participants. Therefore, RR extraction does not introduce any data leakage between training and test sets. All normalization parameters for RR features are estimated using only the training subset within each fold and applied unchanged to validation and test subsets, consistent with the approach used for spectrogram normalization. RR was computed for each of the 20 test cases, and the resulting 20 values were concatenated to form a fixed-length feature vector per subject.

### Model Architecture

**Figure 1.**
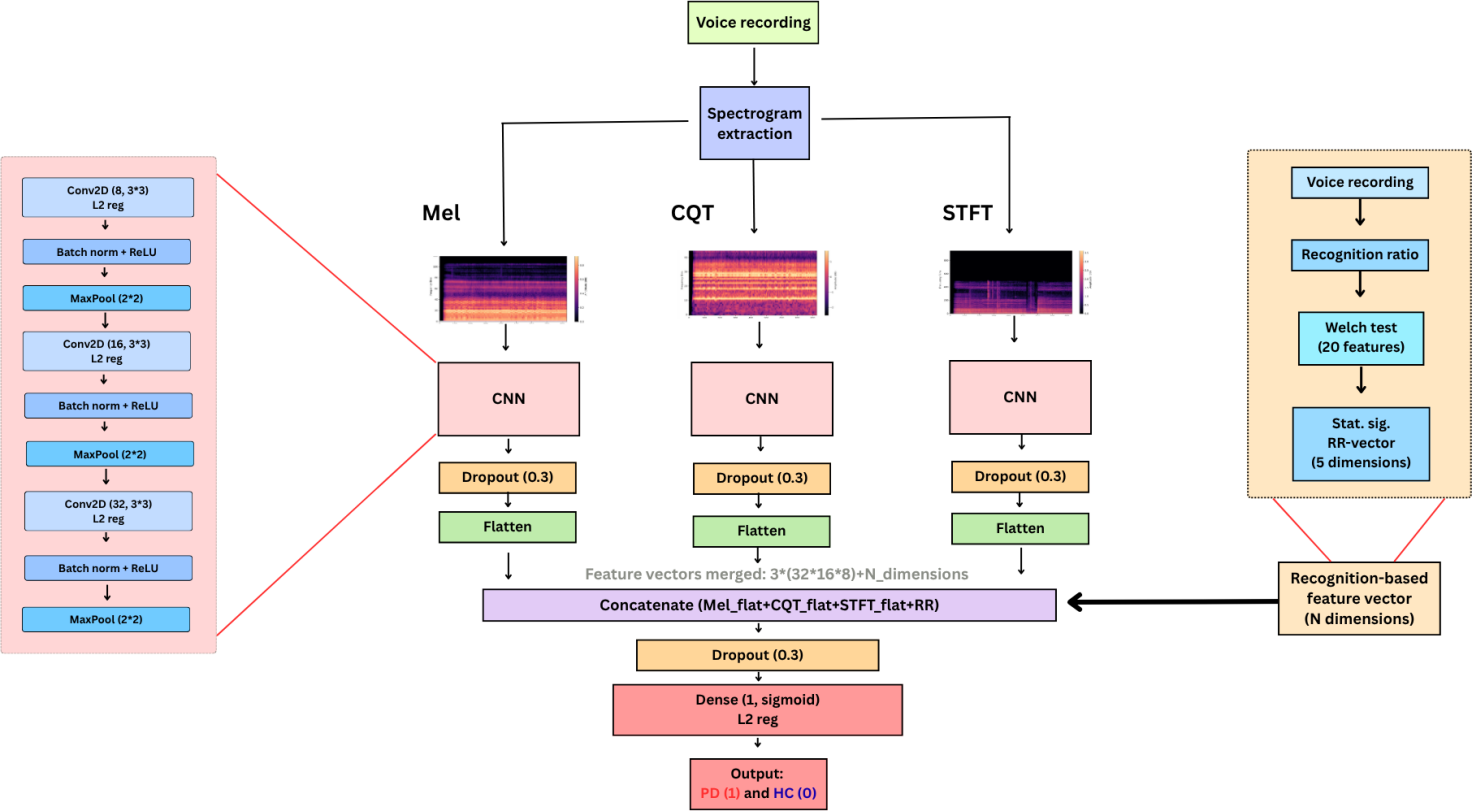
Complete architecture of the proposed multiview spectrogram recognition-aware Parkinson detection network. CNN: convolutional neural network; CQT: constant-Q transform; HC: healthy control; L2 reg: L2 regularization; MaxPool: max pooling; PD: Parkinson disease; ReLU: rectified linear unit; RR: recognition ratio; Stat. Sig.: statistically significant; STFT: short-time Fourier transform.

To accurately classify Parkinson disease through recorded voice, we propose the multiview spectrogram recognition-aware Parkinson detection network (MSR-PDNet), which combines a multibranch CNN with a recognition-aware component derived from the RR. As illustrated in Figure 1, MSR-PDNet receives 3 normalized spectrogram images (Mel, CQT, and STFT) as parallel inputs. Each branch applies stacked 2-dimensional convolutional blocks (convolution, batch normalization, nonlinear activation, and pooling), followed by global pooling to produce a fixed-length embedding vector. The 3 embedding vectors are concatenated into a fused multispectrogram representation, followed by batch normalization and a compact classification head. The output uses a single sigmoid activation to produce the Parkinson disease probability *P*(PD | *x*) ∈ [0*,* 1]. In the full MSR-PDNet model, the RR feature vector is concatenated with the fused spectrogram representation before the classification head, providing lightweight recording-level intelligibility context that complements local spectro-temporal patterns. An ablated variant without RR was also trained under the same protocol.

Models were implemented using TensorFlow and Keras (v2.19.0) and trained with the Adam optimizer at a learning rate of *η*=1×10*^−^*^4^, binary cross-entropy loss, batch size of 16, and dropout of 0.3, for up to 40 epochs. Early stopping required a minimum of 20 epochs and stopped if the validation loss did not improve for 5 consecutive epochs.

### Ethical Considerations

Voice recordings were obtained as part of a prospective clinical study conducted at the Department of Neurology, Inha University Hospital (Incheon, South Korea). The study protocol was approved by the Institutional Review Board of Inha University Hospital (IRB 2022-09-037). All experiments involving human participants were performed in accordance with relevant guidelines and regulations, and written informed consent was obtained from all participants and their legal guardians prior to participation. All collected data were deidentified before analysis. Participants were assigned unique study identification numbers, and only initials and study identification numbers were used during data collection and management. No compensation was provided to participants for their participation in this study.

## Results

This section reports comparative results for MSR-PDNet against traditional machine learning and single-view CNN baselines, together with ablations over spectrogram views and the RR feature.

### Experimental Setup

All models were evaluated using the same 5-fold participant-wise cross-validation protocol. In each fold, all spectrograms and RR feature vectors derived from a given participant were assigned exclusively to either the training or the held-out test set (no participant contributed data to both). For each fold, 80% of participants were used for training and 20% were held out for testing. Within each fold, class imbalance was addressed by bootstrap oversampling of the minority class in the training split only; the held-out test split remained unchanged. All experiments were executed on an Apple Silicon workstation (Apple M4).

### Performance Metrics

Performance was evaluated on the held-out 20% test split using accuracy, precision, recall (sensitivity), *F*_1_-score, and the area under the receiver operating characteristic curve. Parkinson disease was treated as the positive class with a decision threshold of 0.5. Standard confusion matrix counts (true positive [TP], true negative [TN], false positive [FP], false negative [FN]) were used to compute accuracy, precision, recall, and *F*_1_-score:


(2) $$Accuracy=\frac{TP+TN}{TP+TN+FP+FN}$$

(3) $$Precision=\frac{TP}{TP+FP}$$

(4) $$Recall=\frac{TP}{TP+FN}$$

(5) $$F_{1}=\frac{2\;(Precision\times Recall)}{Precision+Recall}$$

The false negative rate (FNR) was defined as FNR=1−Recall. Receiver operating characteristic curves were computed by sweeping the decision threshold, and the area under the curve was averaged across folds. All performance metrics are reported as mean (SD) across the 5-fold participant-wise cross-validation to reflect variability across folds.

### Comparison With Traditional Acoustic Feature–Based Models

**Figure 2.**
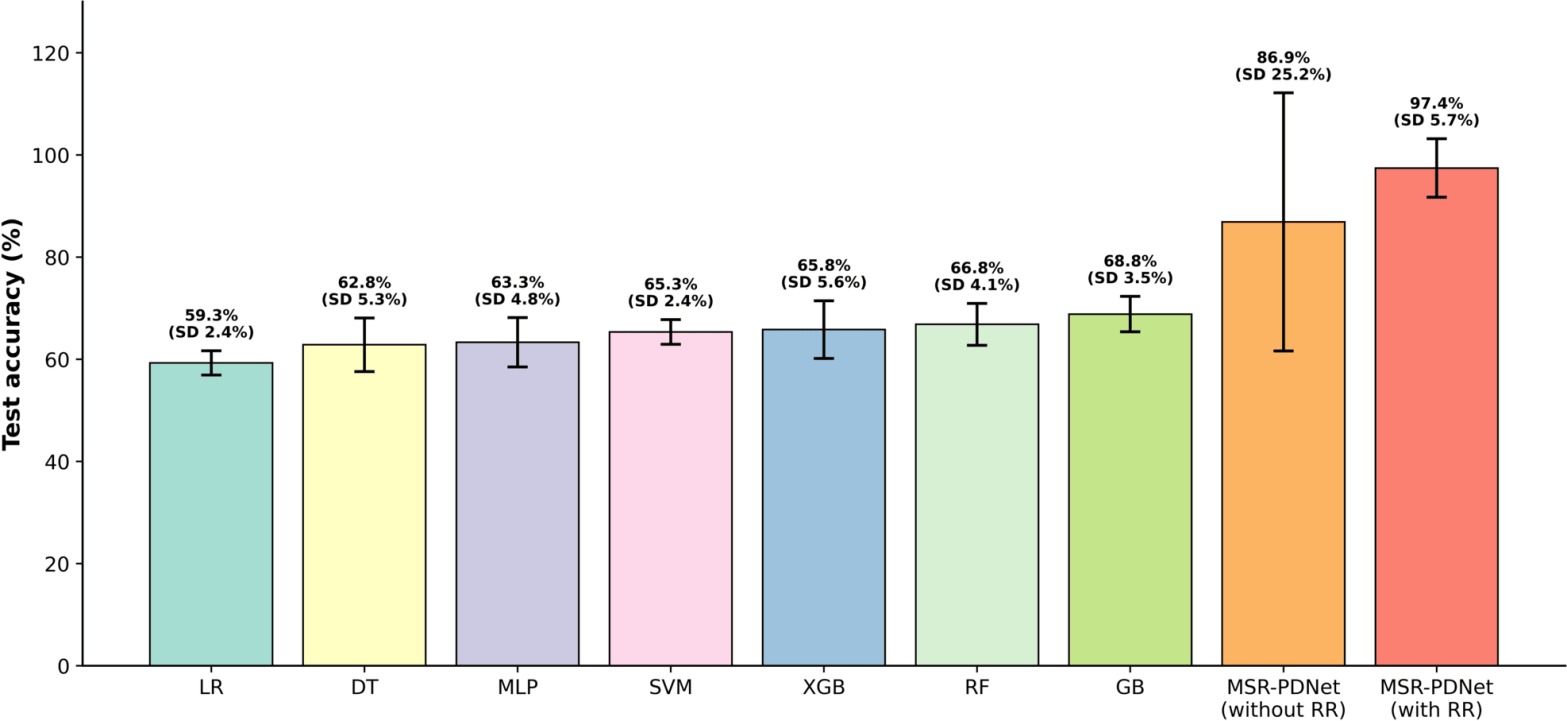
Mean test accuracy comparison between traditional machine learning baselines (acoustic features) and multiview spectrogram recognition-aware Parkinson detection network (with and without RR) under 5-fold cross-validation. DT: decision tree; GB: gradient boosting; LR: logistic regression; MLP: multilayer perceptron; MSR-PDNet: multiview spectrogram recognition-aware Parkinson detection network; RF: random forest; RR: recognition ratio; SVM: support vector machine; XGB: Extreme Gradient BoostIng.

A reference baseline was established using traditional acoustic feature–based machine learning models trained on acoustic descriptors (pitch and loudness statistics, jitter, shimmer, Mel-frequency cepstral coefficient bands 1‐4, and duration) under the same 5-fold participant-wise cross-validation protocol. Mean test accuracy ranged from 59.28% (SD 2.39% for logistic regression) to 68.83% (SD 3.47% for gradient boosting; Table 2; Figure 2).

**Table 2.** Traditional machine learning baselines trained on acoustic features: mean (SD) test accuracy under 5-fold cross-validation.

Model^a^	Test accuracy (%), mean (SD)
Logistic regression	59.28 (2.39)
Decision tree	62.83 (5.26)
MLP^b^	63.32 (4.82)
SVM^c^ (RBF^d^ kernel)	65.35 (2.43)
XGBoost^e^	65.81 (5.64)
Random forest	66.83 (4.10)
Gradient boosting	68.83 (3.47)

a Models are sorted in ascending order of mean test accuracy.

b MLP: multilayer perceptron.

c SVM: support vector machine.

d RBF: radial basis function.

e XGBoost: Extreme Gradient Boosting.

The multilayer perceptron achieved 63.32% (SD 4.82%) mean test accuracy, representing the best-performing configuration under the experimental setup. Key hyperparameters, including the number of hidden layers, neurons per layer, learning rate, activation function, and dropout rate, were systematically tuned using validation performance within each fold. Despite this optimization, the performance of the multilayer perceptron remained lower than that of convolutional models, which may reflect differences in inductive bias. Convolutional architectures are better suited to exploit spatial structure in spectrogram representations, whereas the multilayer perceptron operates on flattened feature vectors.

MSR-PDNet achieved mean test accuracy of 86.9% (SD 25.2%) without RR and 97.4% (SD 5.7%) with RR (Figure 2). The 97.4% result is 38.12 percentage points higher than logistic regression (mean 59.28%, SD 2.39%) and 28.57 percentage points higher than gradient boosting (mean 68.83%, SD 3.47%).

### Ablation Study on Spectrogram Representations

**Figure 3.**
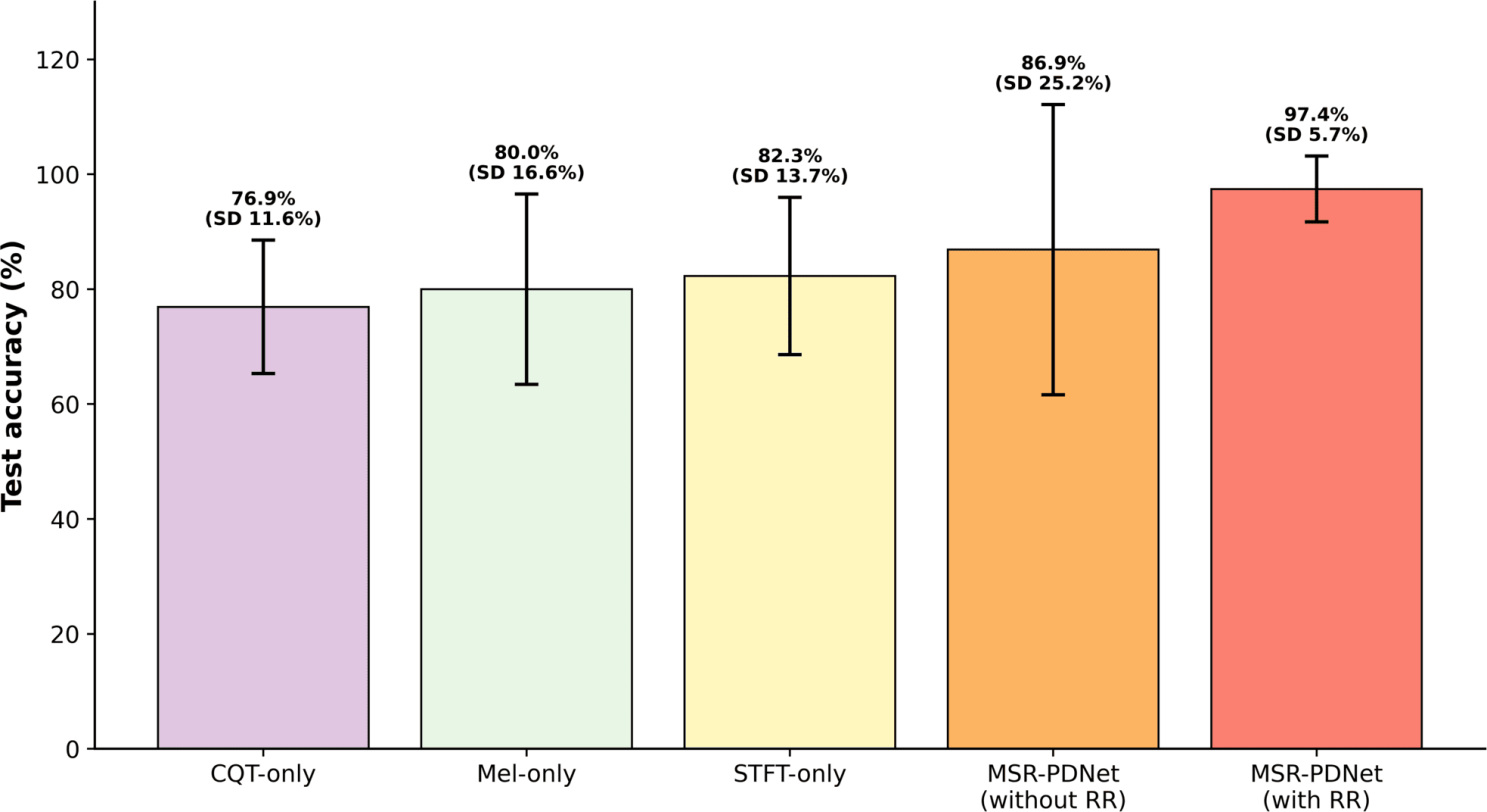
Ablation: mean test accuracy of single-branch baselines and MSR-PDNet (with and without recognition ratio) under 5-fold cross-validation. CQT: constant-Q transform; MSR-PDNet: multiview spectrogram recognition-aware Parkinson detection network; RR: recognition ratio; STFT: short-time Fourier transform.

Ablation results quantify the impact of individual spectrogram views, 2-view fusion, and the RR on classification performance. Single-branch CNN baselines achieved mean test accuracies of 82.3% (SD 13.7% for STFT-only), 80% (SD 16.6% for Mel-only), and 76.9% (SD 11.6% for CQT-only; Table 3; Figure 3). The 3-branch fusion model achieved 86.9% (SD 25.2%) mean test accuracy using spectrograms alone, which is 4.6 percentage points higher than the best single-branch baseline. Incorporating the RR increased accuracy to 97.4% (SD 5.7%; +10.5 percentage points).

**Figure 4.**
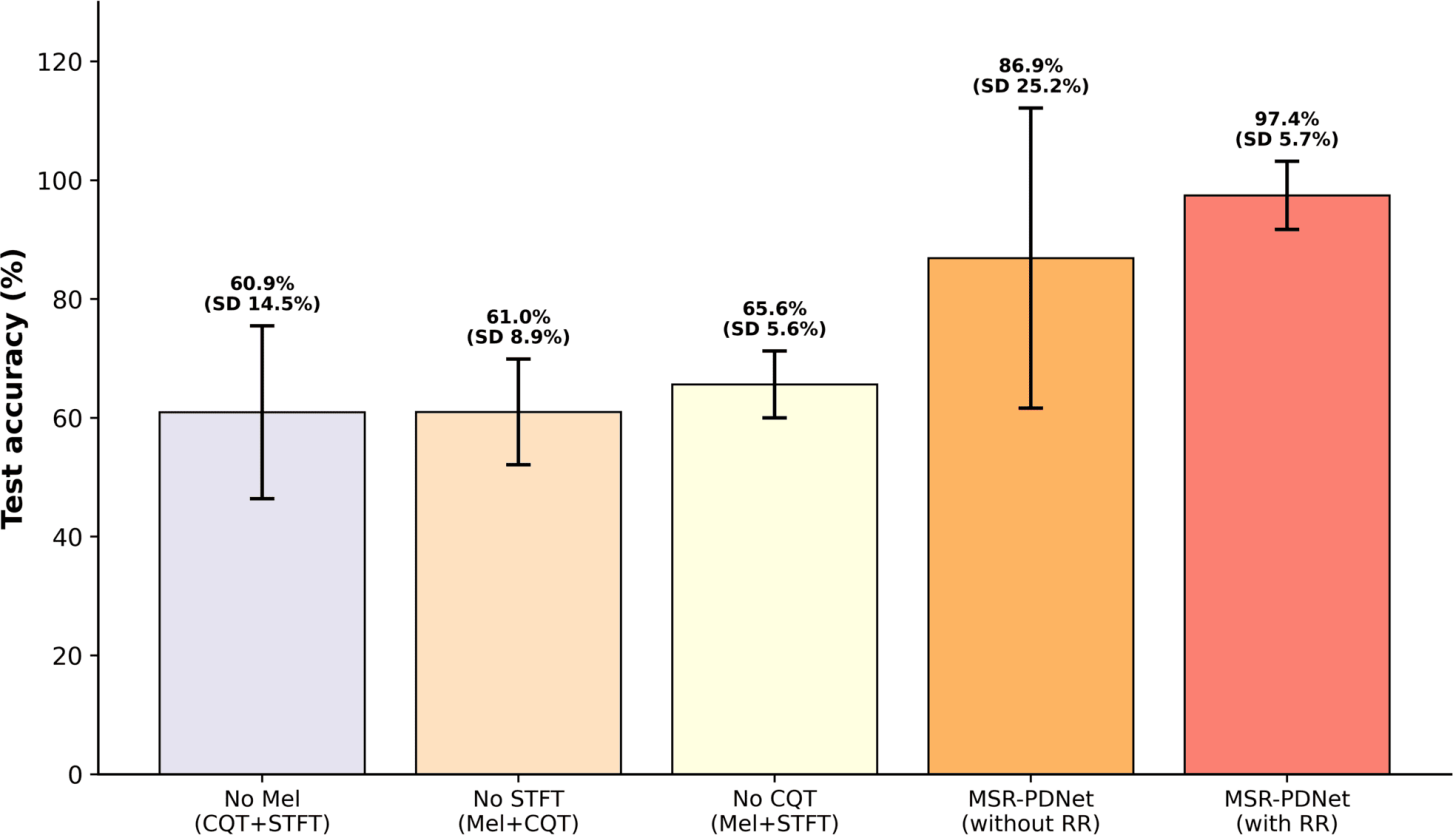
Ablation: mean test accuracy of 2-branch fusion variants and MSR-PDNet (with or without RR) under 5-fold cross-validation. CQT: constant-Q transform; MSR-PDNet: multiview spectrogram recognition-aware Parkinson detection network; RR: recognition ratio; STFT: short-time Fourier transform.

Two-branch ablations yielded 65.6% (SD 5.6%, without CQT; Mel and STFT), 61.0% (SD, 8.9%, without STFT; Mel and CQT), and 60.9% (SD 14.5%, without Mel; CQT and STFT; Table 4; Figure 4).

**Table 3.** Single-branch convolutional neural network baselines and MSR-PDNet variants under 5-fold cross-validation. Results are reported as mean (SD).

Model	Accuracy (%), mean (SD)	Precision (%), mean (SD)	Recall (%), mean (SD)	*F*_1_-score (%), mean (SD)	ROC-AUC^a^ (%), mean (SD)
CQT^b^-only	76.9 (11.6)	85.7 (10)	74.6 (22.2)	77.7 (13.6)	83.2 (8.2)
Mel-only	80 (16.6)	88.1 (7.2)	74.4 (25.1)	79.5 (18.1)	81.9 (12)
STFT^c^-only	82.3 (13.7)	86.9 (8.8)	81 (20.7)	83.1 (14.3)	83.7 (11.8)
MSR-PDNet^d^ (spectrograms only)	86.9 (25.2)	80 (44.7)	77.4 (43.3)	78.6 (42.9)	100 (0.1)
MSR-PDNet (with recognition ratio)	97.4 (5.7)	99 (2.2)	96.5 (7.8)	97.7 (5.2)	98.5 (3.3)

a ROC-AUC: receiver operating characteristic curve-area under the curve.

b CQT: constant-Q transform.

c STFT: short-time Fourier transform.

d MSR-PDNet: multiview spectrogram recognition-aware Parkinson detection network.

**Table 4.** Two-branch ablations in comparison with MSR-PDNet under 5-fold cross-validation.

Model	Accuracy (%), mean (SD)	Precision (%), mean (SD)	Recall (%), mean (SD)	*F*_1_-score (%), mean (SD)	ROC-AUC^a^ (%), mean (SD)
Without CQT^b^ (Mel and STFT^c^)	65.6 (5.6)	85.7 (13.0)	51.9 (12.0)	63.2 (8.2)	84.8 (11.9)
Without Mel (CQT and STFT)	60.9 (14.5)	94.9 (5.0)	35.5 (27.6)	46.6 (28.6)	88.7 (10.5)
Without STFT (Mel and CQT)	61.0 (8.9)	79.8 (10.4)	45.0 (12.9)	56.7 (11.8)	76.9 (14.0)
MSR-PDNet^d^ (with recognition ratio)	97.4 (5.7)	99.0 (2.2)	96.5 (7.8)	97.7 (5.2)	98.5 (3.3)

a ROC-AUC: receiver operating characteristic curve-area under the curve.

b CQT: constant-Q transform.

c STFT: short-time Fourier transform.

d MSR-PDNet: multiview spectrogram recognition-aware Parkinson detection network.

Notably, the spectrogram-only MSR-PDNet configuration showed substantially higher variability across folds (mean 86.9%, SD 25.2%) than the RR-augmented model (mean 97.4%, SD 5.7%), indicating that the recognition-aware feature improves not only accuracy but also stability under participant-wise cross-validation.

### Effect of Recognition-Aware Information on Parkinson Disease Classification

**Figure 5.**
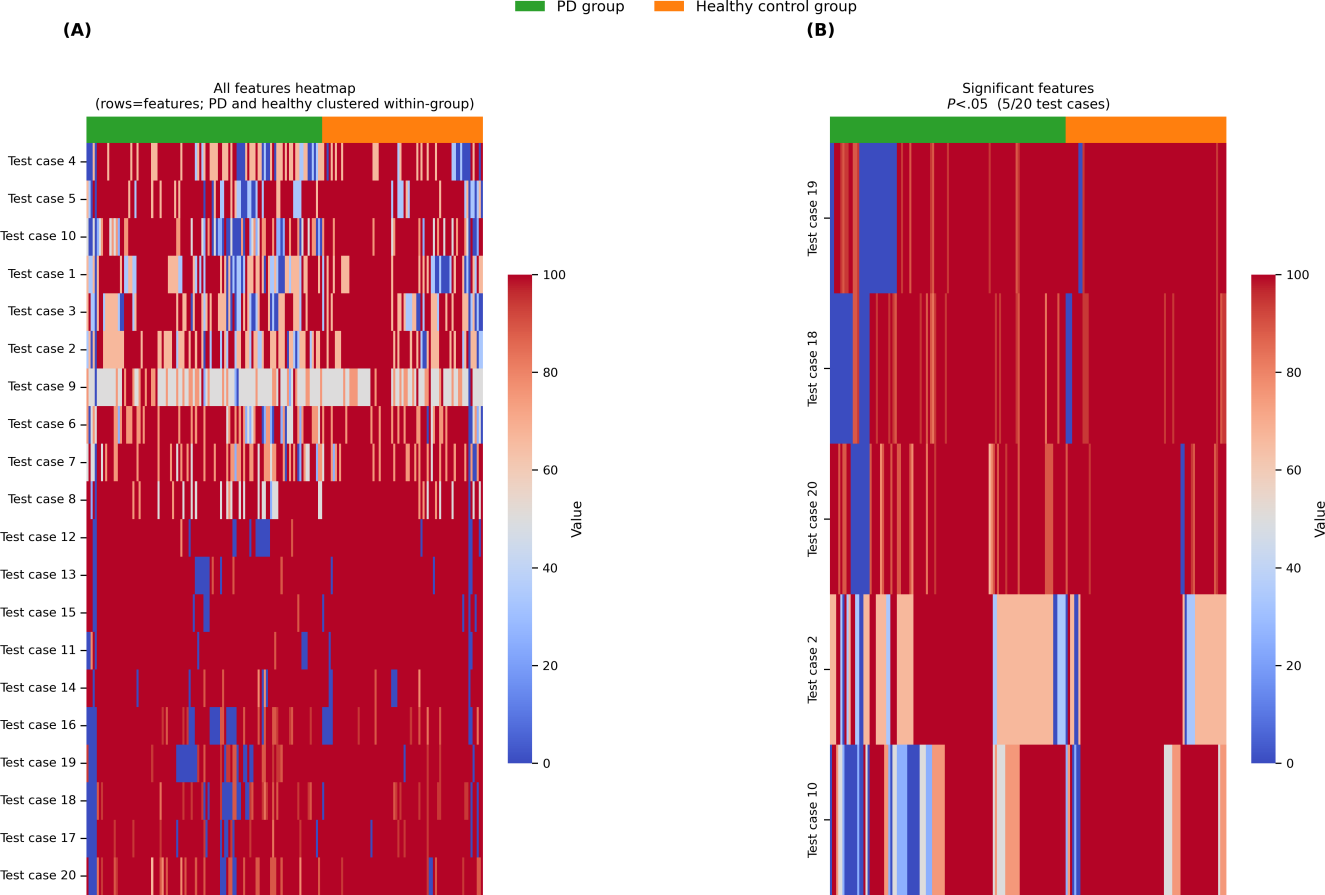
Recognition ratio heatmaps. (A) All test cases and (B) statistically significant test cases (*P*<.05; 5/20). Top annotation indicates Parkinson disease (green) and healthy controls (orange). PD: Parkinson disease.

**Figure 6.**
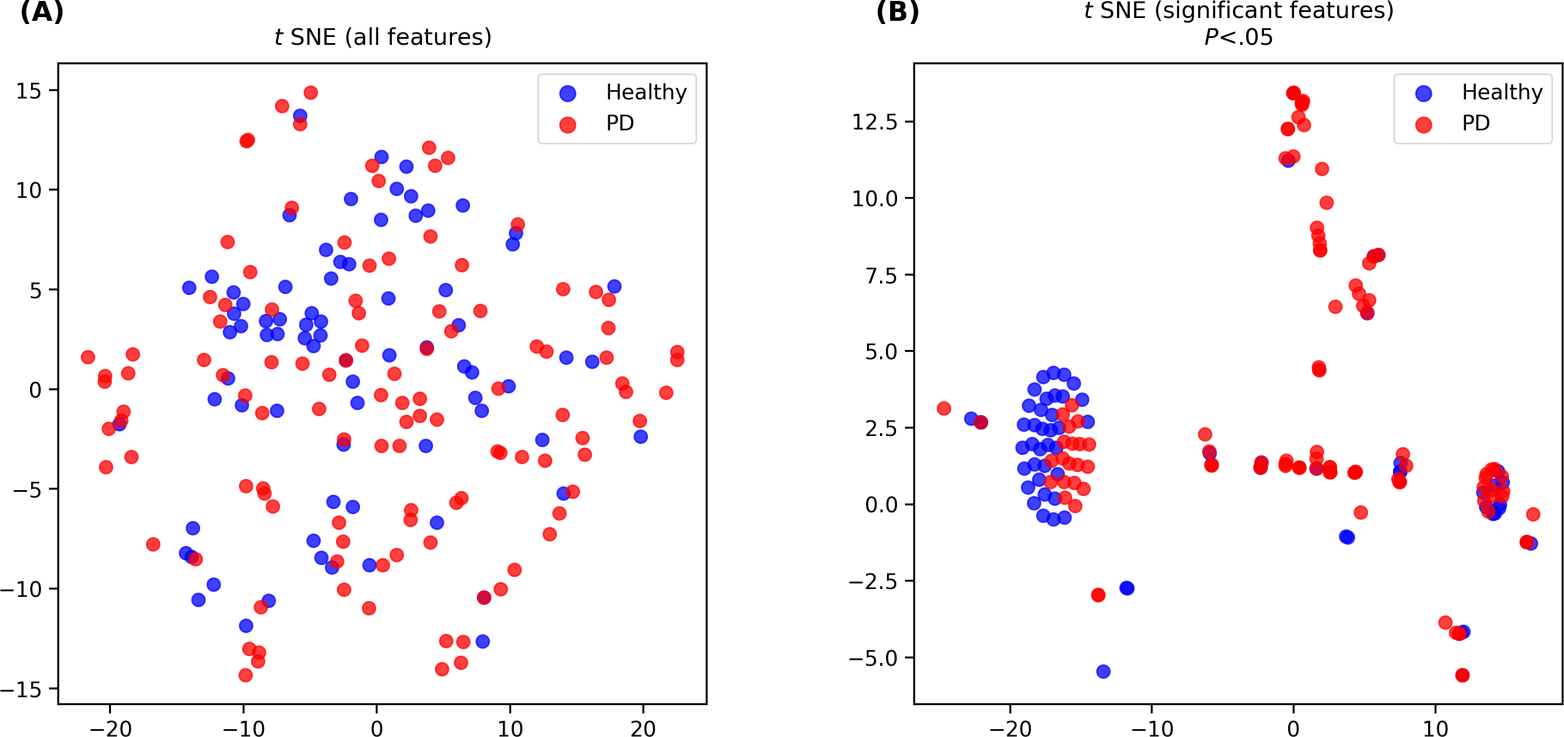
*t* SNE visualizations of the recognition ratio feature space. (A) All test cases and (B) statistically significant test cases. *t* SNE: *t*-distributed stochastic neighbor embedding. PD: Parkinson disease.

To examine whether RR differs between groups, the Parkinson disease and healthy control groups were compared using Welch 2-tailed *t* test independently for each of the 20 test cases. Five test cases showed statistically significant differences at *P*<.05: test cases 2, 10, and 18‐20 (Table 5). Two test cases (10 and 19) remained significant after Bonferroni correction. Concentrating on significant test cases increased the Dunn index from 0.0570 (all test cases) to 0.1640 (significant test cases only), corresponding to a 2.88× increase in cluster separability (Figure 5; Figure 6).

**Table 5.** Recognition ratio test cases showing statistically significant differences between Parkinson disease and healthy control groups.

Test case	*t* test (*df*)^a^	*P* value
Test case 2	−2.103 (162.7)	.04
Test case 10	−4.411 (187.9)	<.001
Test case 18	−2.798 (182.2)	.006
Test case 19	−3.988 (161.9)	<.001
Test case 20	−2.366 (185.0)	.02

a A 2-tailed Welch *t* test was used. A negative *t* indicates a lower mean recognition ratio in the Parkinson disease group. Nominal significance: *P*<.05.

Incorporating RR increased the mean test accuracy from 86.9% (SD 25.2%) to 97.4% (SD 5.7%; Table 3). The mean FNR decreased from 0.226 to 0.035, yielding an approximate type II error reduction:


(6) $$\Delta_{type\;II}(\%)=\frac{FNR_{no\;RR}-FNR_{RR}}{FNR_{no\;RR}}\times 100\approx 84.5\%$$

**Figure 7.**
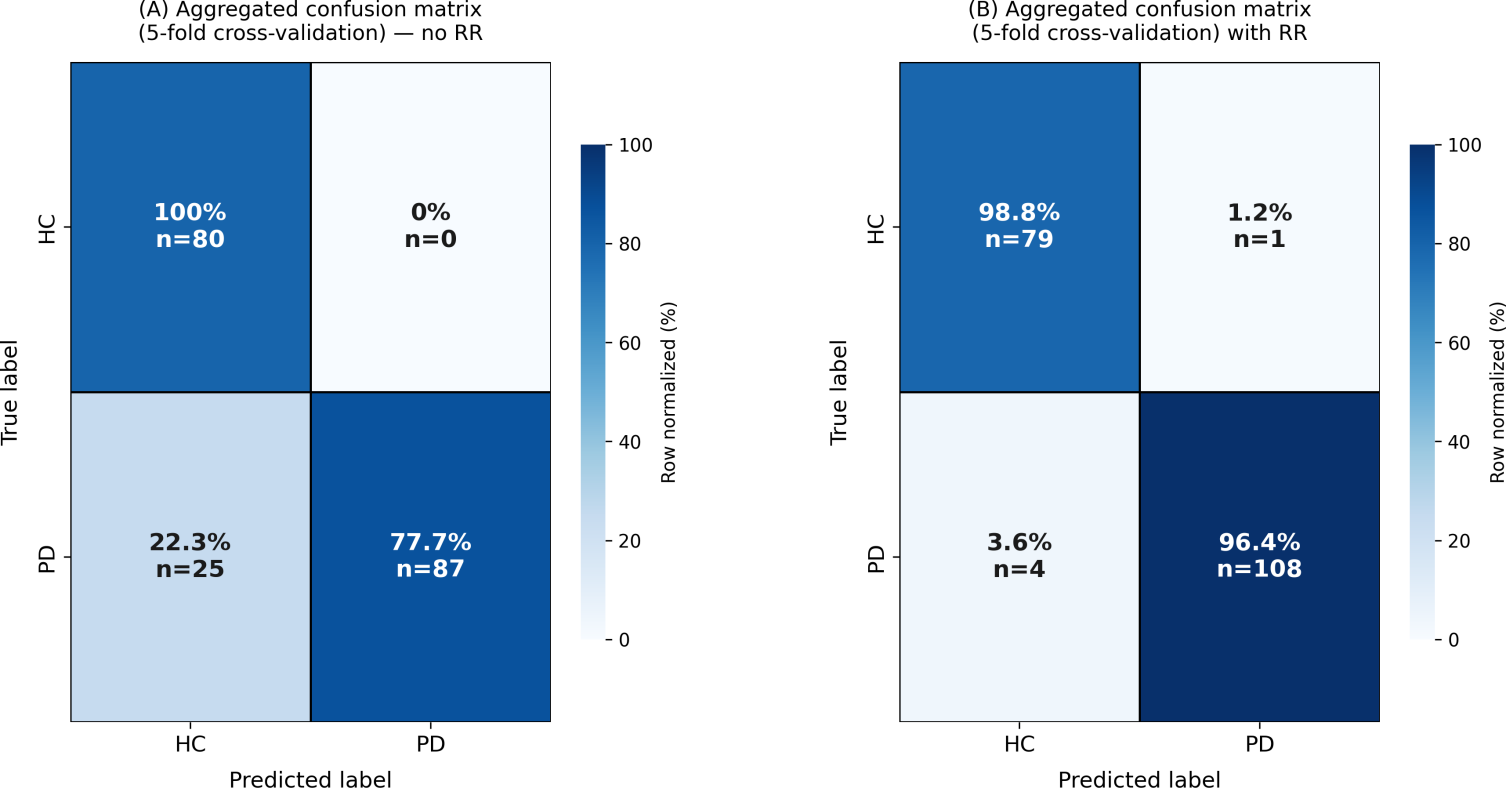
Aggregated 5-fold test confusion matrices for multiview spectrogram recognition-aware Parkinson detection network without RR (A) and with RR (B). Matrices reflect 192 of 203 enrolled participants evaluated at test time (112 with PD and 80 HCs). Eleven participants with missing or unusable recordings were excluded prior to training. Row percentages are computed from pooled counts across all 5 folds. HC: healthy control; PD: Parkinson disease; RR: recognition ratio.

Aggregated confusion matrices are shown in Figure 7.

## Discussion

### Principal Findings

MSR-PDNet integrates 3 complementary spectrogram representations (Mel, STFT, and CQT) with an RR feature vector derived from the same voice recording to support noninvasive Parkinson disease screening. Under strict participant-wise 5-fold cross-validation, 3 main outcomes were observed: spectrogram-based CNN models outperformed traditional acoustic feature machine learning baselines; 3-view spectrogram fusion improved spectrogram-only performance beyond single-view baselines; and integrating the RR produced the largest gain. Mean accuracy increased from 86.9% (SD 25.2%) to 97.4% (SD 5.7%) with RR (+10.5 percentage points), and mean recall increased from 77.4% (SD 43.3%) to 96.5% (SD 7.8%; +19.1 percentage points), reducing the mean FNR from 0.226 to 0.035.

Spectrogram-based CNN models achieved higher held-out accuracy than acoustic feature machine learning baselines under the same protocol. The best single-view baseline (STFT only) reached 82.31% (SD 13.69%), whereas the strongest acoustic feature baseline (gradient boosting) reached 68.83% (SD 3.47%), a 13.47 percentage-point difference. This gap is consistent with time-frequency representations preserving local spectro-temporal patterns that are attenuated when recordings are compressed into summary acoustic statistics.

Three-view spectrogram fusion provided the strongest spectrogram-only performance (mean 86.9%, SD 25.2%), exceeding the best single-view baseline by 4.6 percentage points. Two-view configurations achieved only 60.9% (SD 14.5%) to 65.6% (SD 5.6%), indicating that fusion benefits depend on the specific view combination. Among 2-view configurations, removing Mel features yielded the lowest accuracy, indicating that Mel representations contribute important complementary information consistent with prior Parkinson disease voice classification studies [37-39].

The strong performance of MSR-PDNet (97.4% [SD 5.7%] with RR) likely reflects the complementarity of the proposed feature representation. The 3 spectrogram views (Mel, STFT, and CQT) capture different acoustic aspects of the same voice signal, while the RR provides recording-level information that is not explicitly encoded in local spectrogram patterns. This interpretation is supported by the ablation results: single-view baselines achieved 76.9% (SD 11.6%) to 82.3% (13.7%), 3-view fusion reached 86.9% (SD 25.2%), and adding RR further increased accuracy to 97.4% (SD 5.7%). At the same time, the controlled recording environment, fixed device, and structured reading task may also have contributed to the observed performance.

From a clinical perspective, the proposed model likely captures speech abnormalities associated with hypokinetic dysarthria, a motor speech disorder affecting up to 90% of individuals with Parkinson disease and characterized by reduced vocal loudness, monotonic pitch, imprecise consonant articulation, and diminished speech intelligibility [7,40]. These impairments arise from the progressive degeneration of dopaminergic pathways that govern laryngeal, respiratory, and articulatory musculature, and they constitute some of the earliest detectable nonmotor biomarkers of the disease [8,41]. The 3 spectrogram branches of MSR-PDNet are architecturally aligned with distinct acoustic manifestations of these clinical symptoms. The STFT branch operates on short-time Fourier representations and is particularly sensitive to rapid spectral fluctuations, including vocal tremor, fundamental frequency (F0) instability, and aperiodic noise components that characterize impaired phonation in Parkinson disease [7,42]. The Mel-scale branch maps frequency content onto a perceptually weighted scale that emphasizes the low-to-mid frequency range, making it well-suited to detecting reduced vocal loudness, disrupted harmonic structure, and the breathy voice quality associated with hypophonia in Parkinson disease [22,41]. The CQT branch provides logarithmic frequency resolution with high spectral precision at lower frequencies, enabling the model to identify monopitch patterns, harmonic distortion, and reduced vowel space that correspond to the monotone speech and imprecise articulation characteristic of hypokinetic dysarthria [40,43]. By fusing these 3 complementary representations, MSR-PDNet captures a broader and more clinically complete acoustic profile of Parkinson disease–related vocal impairment than any single-branch spectrogram could encode independently, which is directly consistent with the ablation results, showing that removing any single view substantially reduces performance.

Beyond vocal spectrograms, the RR feature incorporated in the full MSR-PDNet model reflects a clinically distinct dimension of Parkinson disease pathophysiology: respiratory motor dysfunction. A recent meta-analysis confirmed significantly elevated resting respiratory rates in Parkinson disease patients compared with healthy controls [44], and a large-scale clinical study reported a 44% prevalence of respiratory dysfunction in Parkinson disease, attributing it to impaired thoracic musculature control and reduced respiratory drive [45]. The RR, derived from sentence-reading performance, encodes both articulatory precision and respiratory support for speech, thereby providing physiological information that is qualitatively complementary to the spectral features captured by the 3 spectrogram branches. This clinical complementarity explains the substantial accuracy gain observed when the RR is added to the spectrogram-only model (mean 86.9%, SD 25.2% to mean 97.4%, SD 5.7%): the 2-feature modalities jointly reflect the vocal tract impairment and the respiratory motor impairment that together define hypokinetic dysarthria in Parkinson disease. Therefore, the model’s behavior can be interpreted in terms of clinically meaningful manifestations of Parkinson disease–related speech impairment rather than purely abstract signal patterns.

RR integration produced the largest improvement in accuracy and sensitivity. The corresponding FNR decreased from 0.226 to 0.035 (approximately 84.5% reduction), which is relevant for screening-oriented settings where missed cases are clinically undesirable.

Although the RR may be affected by factors such as accent, language proficiency, and recording quality, several findings support its relevance to Parkinson disease–related speech changes. RR differences were observed between relatively age-matched Parkinson disease and healthy control groups (mean age 68.7 [SD 8.9] y vs 65.3 [SD 9.8] y), significant differences were identified across specific sentence-reading tasks (Table 5), and controlled acquisition conditions reduced recording-related variability. Therefore, although RR should not be regarded as a fully disease-specific biomarker in isolation, it appears to provide a meaningful recognition-aware feature reflecting Parkinson disease speech characteristics.

Recent state-of-the-art voice-based Parkinson disease classification studies were reviewed alongside MSR-PDNet, as summarized in Table 6. Given that each study adopts its own dataset, feature extraction approach, and evaluation protocol, the figures presented here serve as a broad reference point rather than a strict performance benchmark.

**Table 6.** Comparison with recent state-of-the-art voice-based Parkinson disease classification methods, including MSR-PDNet (spectrogram only) and MSR-PDNet (spectrogram+RR).

Method	Type	Accuracy (%)	Reference
Vision transformer (ViT+AST^a^)	Single	73	Perrone et al [46]
ResNet CNN^b^	Single	84	Escobar-Grisales et al [47]
EfficientNet-B2	Multi	84.39	Malekroodi et al [48]
MSR-PDNet^c^ (spectrogram only)	Multi	86.9	Ours
DenseNet-161 (TL^d^)	Single	89.75	Karaman et al [49]
VGG-16^e^	Single	91.8	Malekroodi et al [50]
VGG-16	Single	92	Bhatt et al [51]
DenseNet+MobileNet+ShuffleNet	Multi	95.56	Chen et al [26]
CNN-LSTM^f^	Single	95.67	Shibina and Thasleema [52]
MSR-PDNet (spectrogram+RR^g^)	Multi	97.4	Ours

a AST: audio spectrogram transformer.

b CNN: convolutional neural network.

c MSR-PDNet: multiview spectrogram recognition-aware Parkinson detection network.

d TL: transfer learning.

e VGG-16: visual geometry group 16.

f LSTM: long short-term memory.

g RR: recognition ratio.

Among single-spectrogram approaches, reported accuracies ranged from 73% to 84%, with several architectures, including DenseNet-161 (89.75% [49]), visual geometry group 16 (VGG-16: 91.8% [50]), Superlet-based VGG-16 (92% [51]), and CNN and long short-term memory (95.67% [52]), yielding higher values under their own experimental conditions. MSR-PDNet with RR achieved 97.4%, representing a strong result within this broader landscape, although cross-study variability in datasets and evaluation protocols limits direct interpretability.

Similarly, multibranch and multimodel configurations such as EfficientNet-B2 fusion (84.39% [48]) and ensemble models (95.56% [26]) reflect the growing interest in combining multiple representations, which aligns with the motivation behind integrating multiview spectrograms with RR features in the proposed approach.

Despite these promising results, several limitations should be noted. First, evaluation was performed on a single-center dataset collected under controlled conditions using the same recording device and protocol. Accordingly, performance is expected to generalize best to similar clinical settings, whereas different cohorts, devices, and real-world environments may introduce distribution shifts that affect both spectrogram features and RR computation. Future work should therefore include multicenter validation, noise-aware data augmentation, and domain adaptation to improve robustness.

Second, bootstrap oversampling was applied in the training split to address class imbalance; further evaluation of probability calibration would be beneficial for clinically meaningful sensitivity-specificity tradeoffs.

Third, RR depends on the speech task design and the automatic speech recognition pipeline, and it may vary across languages, prompts, and recognition systems.

Fourth, the current system requires a standardized sentence-reading task with predefined prompts to compute the RR, making it most suitable for supervised clinical or at-home screening. Performance may be affected by background noise, device variability, and shorter recordings, and adaptation would be needed for spontaneous-speech settings. Furthermore, the effectiveness of the RR is likely sensitive to the phonetic and articulatory characteristics of the selected prompts. While the current 20-sentence protocol contributed to improved classification performance, systematically optimized sentence designs may further enhance the ability to capture Parkinson disease–related speech impairments. Future work should therefore evaluate robustness under diverse recording conditions, explore more flexible speech protocols, and investigate prompt optimization strategies to maximize the diagnostic sensitivity of the RR feature.

Finally, the low accuracy of 2-view configurations indicates that fusion benefits depend strongly on view pairing and training stability, and broader testing across datasets and alternative fusion strategies is warranted.

### Conclusions

This study presents MSR-PDNet, a multiview spectrogram-based framework for noninvasive Parkinson disease screening from voice recordings. Across participant-wise 5-fold cross-validation, the method achieved 86.9% (SD 25.2%) mean test accuracy using spectrogram fusion, improving to 97.4% (SD 5.7%) when the RR vector was added. The RR-augmented model showed higher sensitivity (recall: mean 96.5%, SD 7.8%) compared with the spectrogram-only configuration (mean 77.4%, SD 43.3%), with a substantially reduced FNR relevant for screening. Comparison with recent state-of-the-art methods suggested that MSR-PDNet is competitive within the current literature. Future work will focus on external validation, robustness testing across multidevice and multicenter cohorts, and adaptation to more flexible recording conditions to support clinical translation.

## Supplementary material

10.2196/94063 Multimedia Appendix 1 Detailed spectrogram normalization equations.

## Abbreviations

CNN: convolutional neural network
CQT: constant-Q transform
FN: false negative
FNR: false negative rate
FP: false positive
MSR-PDNet: multiview spectrogram recognition-aware Parkinson detection network
RR: recognition ratio
STFT: short-time Fourier transform
TN: true negative
TP: true positive
VGG-16: visual geometry group 16
