# Supplementary material for: A Machine Learning Approach to Voice-Based Parkinson Disease Screening Using Multiview Spectrogram and Speech Recognition Features: Diagnostic Study
Source: JMIR Med Inform. 2026 Jun 11;14:e94063. doi: 10.2196/94063 (PMC13255941; doi:10.2196/94063)
Supplement: Multimedia Appendix 1 [file medinform-v14-e94063-s001.docx]

# Multimedia Appendix 1: Detailed Spectrogram Normalization Equations

This appendix provides the detailed equations for the three spectrogram normalization procedures applied in the preprocessing pipeline. All normal- ization parameters were estimated using only the training subset within each fold and applied unchanged to validation and test subsets, preventing data leakage.

**Mel spectrogram normalization.** The log-Mel spectrogram *S*_Mel_ and its fold-wise min–max normalized form *S*˜Mel are defined as:

*
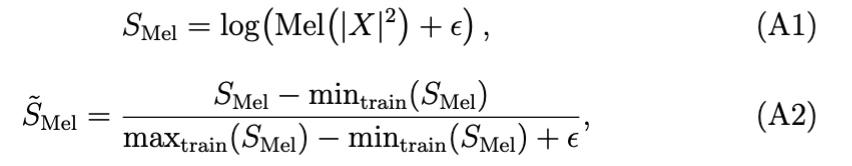
S*_Mel_ = log Mel |*X*|^2^ + *ϵ* *,* (A1)

*S*Mel − mintrain(*S*Mel)

˜

*S* = *,* (A2)

Mel

maxtrain

(*S*Mel

) − min

train

(*S*Mel

) + *ϵ*

where *X* = STFT(*x*) is the short-time Fourier transform of waveform *x*,

|*X*|^2^ is the power spectrum, Mel(·) denotes the Mel filterbank mapping, and

*ϵ* = 10*^−^*^8^ ensures numerical stability.

**Constant-Q transform spectrogram normalization.** The log-CQT spectrogram *S*_CQT_ and its z-score normalized form *S*˜CQT are:

*
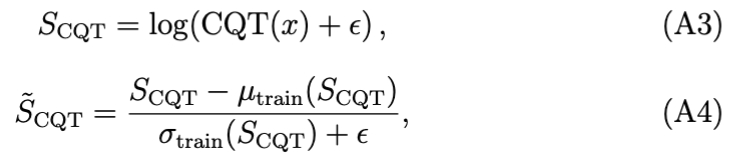
*

*S*_CQT_ = log(CQT(*x*) + *ϵ*) *,* (A3)

*S*˜ = *S*CQT − *µ*train(*S*CQT) *,* (A4)

CQT

*σ*train

(*S*CQT

) + *ϵ*

where CQT(·) is the constant-Q transform magnitude and *µ*_train_(·), *σ*_train_(·) are element-wise mean and standard deviation on the training subset.

**Short-time Fourier transform spectrogram normalization.** The log-magnitude STFT spectrogram *S*_STFT_ and its robust-scaled form *S*˜STFT are:

*
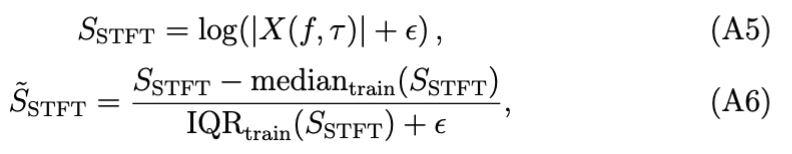
*

*S*_STFT_ = log(|*X*(*f, τ* )| + *ϵ*) *,* (A5)

*S*˜ = *S*STFT − mediantrain(*S*STFT) *,* (A6)

STFT

IQR

train

(*S*STFT

) + *ϵ*

where *X*(*f, τ* ) is the complex short-time Fourier transform at frequency bin *f* and time frame *τ* , and median_train_(·), IQRtrain(·) are element-wise median and interquartile range computed on the training subset.
